# Mental Health Symptoms and Receipt of Mental Health Care Among US Adults Diagnosed With Kidney Disease

**DOI:** 10.5888/pcd22.240509

**Published:** 2025-07-03

**Authors:** Maria A. Villarroel, Xun Wang

**Affiliations:** 1 Division of Health Interview Statistics, National Center for Health Statistics, Centers for Disease Control and Prevention, Hyattsville, Maryland

## Abstract

**Introduction:**

Nationally representative estimates of mental health symptoms and services in adults with kidney disease are limited. The objective of this study was to examine the mental health status and use of health care among adults with and without kidney disease.

**Methods:**

We used data from the 2021 National Health Interview Survey. Diagnosed kidney disease is based on adults who reported ever being told by a doctor or other health professional that they had weak or failing kidneys. The survey question captures data on adults who are aware of having kidney disease and most likely have advanced kidney disease. Mental health measures examined were serious psychological distress (SPD), current symptoms of anxiety and depression, diagnosed anxiety and depressive disorder, prescription medication use for these disorders, and receipt of counseling. We used logistic regression models, with predicted marginal proportions, to calculate unadjusted and adjusted prevalence ratios, controlling for sociodemographic and health characteristics.

**Results:**

About 2.9% of adults reported having a diagnosis of kidney disease; prevalence varied by sociodemographic and health characteristics. The prevalence of SPD; current symptoms of anxiety or depression or both; history of diagnosed anxiety or depression or both; and receiving counseling and prescription use for these disorders were higher among adults with kidney disease than among adults without kidney disease. In multivariable models adjusted for sociodemographic and health characteristics, adults with diagnosed kidney disease remained more likely than adults not diagnosed with kidney disease to experience mental health conditions and receive counseling.

**Conclusion:**

A survey of the US population found a higher prevalence of poor mental health and receipt of mental health care among people diagnosed with kidney disease than among people not diagnosed with kidney disease.

**Summary**

**What is already known on this topic?**

Studies conducted in clinical settings indicate that depression and anxiety may be common among patients with kidney disease.

**What is added by this report?**

Data from the 2021 National Health Interview Survey showed that adults with diagnosed kidney disease were more likely than adults without diagnosed kidney disease to experience symptoms of serious psychological distress, feelings of anxiety that can affect functioning, have a history of diagnosed anxiety and depressive disorders, and to have received counseling service, after adjusting for sociodemographic and other health characteristics.

**What are the implications for public health practice?**

This analysis enhances our understanding of the intersection between diagnosed kidney disease and mental health conditions.

## Introduction

Kidney disease is a leading cause of death in the US (1). The associations between mental health conditions and kidney disease may impair people’s functioning, increase risk of self-harm, and affect the ability to deal with stressors and medical care demands of their conditions (2–5). Studies conducted in clinical settings using various mental health assessment tools have found that depression and anxiety may be common in patients with kidney disease (6–11). However, population-based studies examining mental health status in people with kidney disease are limited (12).

Mental health distress in people with kidney disease is thought to be the result of multiple cascading factors — decreased renal function may affect neurohormonal activity and immunological responses reflected in somatic symptoms and feelings of sickness (eg, sleep disturbance, fatigue, decreased sexual drive) (6,8,10,11,13). Biological factors and physical effects of kidney disease may also interrelate with interpersonal factors (eg, changes in social roles; limited capacity for self-expression, productivity, and social involvement) and with socioeconomic factors such as low income or job loss that may contribute to psychological distress and worsening health (6). Patients with kidney disease may also face multiple stressors as the condition, treatment, and recommended lifestyle changes threaten their feelings of independence, autonomy, and achievement in everyday life, and with advancing stage of disease, the awareness of being at high risk of death (6). Research on the receipt of mental health care among people with kidney disease is limited (7,12,14).

The primary objectives of this study were to examine diagnosis history for depressive and anxiety disorders among adults who have been diagnosed with kidney disease and those who have not and current mental health symptomology that may affect their functioning and require treatment. This study also examines the use of prescription medication for depression and anxiety and the receipt of counseling services.

## Methods

We used data from the 2021 National Health Interview Survey (NHIS) to generate prevalence estimates (15). NHIS is a nationally representative household health survey of the civilian noninstitutionalized US population. It is conducted continuously throughout the year by the National Center for Health Statistics (NCHS). Interviews are typically initiated face-to-face in respondents’ homes with follow-ups conducted by telephone as needed. One “sample adult” aged 18 years or older and 1 “sample child” aged 17 years or younger (if any children live in the household) are randomly selected from each household to provide information about various health topics. Information about adults is self-reported. If the sample adult is physically or mentally unable to self-report, a knowledgeable proxy can answer for them. During 2021, the NHIS collected data for 29,482 sample adult interviews; the response rate was 49.9%. About 2.0% or 582 sample adult interviews were from a proxy respondent; most proxy respondents (95.0%) identified as a relative of the sample adult.

### Measures

We identified adults diagnosed as having kidney disease from a question that asked about ever being told by a doctor or other health professional that they had weak or failing kidneys; respondents were asked not to include kidney stones, bladder infections, or incontinence. This question has also been used to assess awareness of chronic kidney disease in adults with estimated glomerular filtration rate and urine albumin-to-creatine ratio tests (16). During 2017–2020, 25.6% of adults in the general population with mildly to moderately decreased function to kidney failure (stages 3a–5 in the Kidney Disease Outcomes Quality Initiative staging guidelines of chronic kidney disease) reported having been told they had kidney disease (16). Awareness of kidney disease increased with disease severity from 16.8% among those in stage 3a to 83.9% among those in stage 5 of chronic kidney disease (16). The NHIS collects information about kidney disease every 3 years; it does not collect information about kidney functioning, disease duration, or biomarker data. The findings from adults diagnosed as having kidney disease in this study are more likely to reflect those who have more advanced kidney disease and are aware of their kidney disease.

Measures of mental health status were ever diagnosed by a doctor or health care professional with depressive disorder, anxiety disorder, or both, and 2 sets of questions about current symptomology. The first is a measure of serious psychological distress (SPD) based on the Kessler 6 (K6) scale (17,18). This is a validated population-based screener designed to identify people with and without symptoms of serious mental illness, defined as a severity of symptoms that may cause impairment in social and occupational functioning and require treatment. The K6 scale (17) is significantly correlated with measures that predict several anxiety and mood disorders from the *Diagnostic and Statistical Manual of Mental Disorders, Fourth Edition.* The second set of current symptoms measures are based on the psychosocial functional domain from Washington Group on Disability Statistics (19). The Washington Group on Disability Statistics focuses on several domains of functional difficulties, which are designed to identify people who are at increased risk of experiencing limited or restricted participation in society. The psychosocial domain questions measure frequency and intensity of feelings of anxiety and depression. These measures are not diagnostic but have a high degree of agreement with scales that identify moderate to severe symptoms of generalized anxiety disorder and is diagnostic of depression (20). The wording of measures from the 2021 NHIS related to mental health status and care, sociodemographic characteristics, and health conditions is provided (Supplemental Table). Measures of mental health care included use of prescription medication for anxiety or depression or both and receiving counseling or therapy from a mental health professional, such as a psychiatrist, psychologist, psychiatric nurse, or clinical social worker, in the past 12 months and currently.

We included several sociodemographic and health characteristics associated with diagnosed kidney disease and mental health conditions as confounders based on a priori criteria. Sociodemographic measures were sex, age, race and Hispanic origin, education level, marital status, family income as a percentage of federal poverty level, with imputed values when data were missing (15,21), and urbanization levels (22). Selected health characteristics were self-reported health status, categorized into fair or poor versus excellent, very good, or good; and having been diagnosed by a doctor or other health professional with the following conditions: ever diagnosis of coronary heart disease, diabetes, hyperlipidemia, and hypertension, where diagnosis of hyperlipidemia and hypertension was limited to having the condition in the past 12 months.

### Analyses

Statistical analyses were weighted to account for the complex survey design of the NHIS. We used SAS-callable SUDAAN version 11.0 (RTI International) to perform all analyses. We calculated prevalence estimates of US adults diagnosed with kidney disease by selected sociodemographic and health characteristics, mental health status, and receipt of mental health care; we used the Korn–Graubard method for complex surveys to generate 95% CIs. All estimates presented meet NCHS data presentation standards for proportions (23). We used Wald *F* tests to test for any differences in diagnosed kidney disease across sociodemographic characteristic, health characteristic, and mental health characteristic, excluding missing or unknown responses. We evaluated linear trends by age group, education, family income, and urbanization level by using orthogonal polynomials in logistic regression (24).

We used logistic regression models, with predicted marginal proportions (25), to calculate unadjusted prevalence ratios (PRs) and 2 separate adjusted PRs (APRs) examining the association between diagnosed kidney disease and selected measures of mental health status and care. The first adjusted model controlled only for sociodemographic characteristics: sex, age, race and Hispanic origin, family income, education, marital status, and urbanization level. The second adjusted model controlled for sociodemographic characteristics from the first model and added health characteristics: self-reported health status, ever diagnosis of diabetes and coronary heart disease, and ever diagnosis of hypertension and hyperlipidemia and having it in the past 12 months.

## Results

In 2021, 2.9% of adults aged 18 years or older had been diagnosed with kidney disease (Table 1). The prevalence of adults diagnosed with kidney disease did not differ between men and women, but it significantly differed by other sociodemographic characteristics including age, race and Hispanic origin, marital status, and urbanization level. The prevalence of diagnosed kidney disease was higher among adults with self-reported fair or poor health (vs excellent, very good, or good health) and among adults with (vs without) other diagnosed chronic conditions.

**Table 1:** Percentage of Adults Diagnosed With Kidney Disease, by Selected Sociodemographic and Health Characteristics, National Health Interview Survey, 2021^a^

Characteristic	% (95% CI)	*P* value, Wald *F* test^b^	*P* value, linear trend^c^
**Total**	2.9 (2.7–3.1)	—	NA
**Sex**			
Men	2.8 (2.5–3.2)	.88	NA
Women	2.9 (2.6–3.1)		
**Race and Hispanic origin^d^**			
Hispanic	2.6 (2.1–3.2)	.01	NA
Non-Hispanic Asian	1.8 (1.1–2.6)		
Non-Hispanic Black	3.3 (2.7–4.0)		
Non-Hispanic White	2.9 (2.7–3.2)		
**Age, y**			
18–44	0.9 (0.7–1.1)	<.001	<.001
45–64	2.9 (2.5–3.3)		
≥65	6.8 (6.2–7.4)		
**Education**			
High school diploma or less	4.0 (3.6–4.4)	<.001	<.001
Associate’s degree or some college	3.0 (2.6–3.4)		
Bachelor’s degree or higher	1.6 (1.4–1.9)		
**Marital status**			
Married	2.6 (2.3–2.9)	<.001	NA
Separated, divorced, or widowed	6.1 (5.5–6.8)		
Never married	1.5 (1.2–1.9)		
Cohabiting	1.9 (1.3–2.7)		
**Family income, percentage of federal poverty level^e^**			
<100%	4.5 (3.7–5.4)	<.001	<.001
100% to <200%	4.3 (3.7–4.9)		
≥200%	2.3 (2.1–2.5)		
**Urbanization level^f^**			
Large metropolitan area	2.5 (2.2–2.8)	<.001	<.001
Medium or small metropolitan area	3.2 (2.8–3.5)		
Nonmetropolitan area	3.8 (3.1–4.5)		
**Self-reported health status**			
Excellent, very good, or good	1.6 (1.4–1.7)	<.001	NA
Fair or poor	11.2 (10.1–12.3)		
**Ever diagnosed with diabetes**			
Yes	11.1 (9.9–12.4)	<.001	NA
No	2.0 (1.8–2.2)		
**Ever diagnosed with coronary heart disease**			
Yes	13.1 (11.3–15.1)	<.001	NA
No	2.3 (2.1–2.5)		
**Hyperlipidemia in the past 12 months**			
Yes	6.7 (6.0–7.3)	<.001	NA
No	1.8 (1.6–2.0)		
**Hypertension in the past 12 months**			
Yes	7.4 (6.8–8.0)	<.001	NA
No	1.2 (1.0–1.4)		

Abbreviation: NA, not applicable.

a The National Health Interview Survey is conducted by the National Center for Health Statistics (15). Diagnosed kidney disease is based on adults who reported ever being told by a doctor or other health professional that they had weak or failing kidneys.

b *P* value from Wald *F* tests, testing for any differences across groups, excluding missing or unknown.

c *P* value from linear trend evaluated using orthogonal polynomials in logistic regression (24).

d Estimates for adults of another race or of multiple races are not shown due to the inability to make statistically reliable estimates for these groups, but they are included in the analyses.

e Imputed when missing (15,21).

f Combined categories from the 2013 National Center for Health Statistics Urban–Rural Classification Scheme for Counties (22).

Adults diagnosed with kidney disease had a significantly higher prevalence of various mental health outcomes compared with those not diagnosed with kidney disease (Table 2). Adults diagnosed with kidney disease were almost 3 times more likely to be experiencing SPD than adults not diagnosed with kidney disease (10.4% vs 3.5%). Compared with adults not diagnosed with kidney disease, adults diagnosed with kidney disease were more likely to have current symptoms of anxiety, to have been diagnosed with an anxiety disorder, to have current symptoms of depression, and to have been diagnosed with a depressive disorder. Adults diagnosed with kidney disease were also more likely to have diagnosed comorbidity of anxiety and depressive disorders and have coexisting symptoms of anxiety and depression. Adults diagnosed with kidney disease were more likely than those not diagnosed with kidney disease to be currently taking medication for feelings of anxiety or depression or both. A higher percentage of adults diagnosed with kidney disease received counseling in the past 12 months and were currently receiving counseling, but these differences were not significant.

**Table 2:** Percentage of Adults With Mental Health Symptoms, History of Mental Health Diagnosis, and Mental Health Care, by Diagnosis of Kidney Disease Condition, National Health Interview Survey, 2021^a^

Characteristic	All	With kidney disease	Without kidney disease	*P* value^b^
**Mental health status**				
Serious psychological distress	3.7 (3.4–4.0)	10.4 (8.0–13.2)	3.5 (3.2–3.8)	<.001
Ever diagnosed with anxiety disorder	16.4 (15.8–16.9)	26.2 (23.0–29.7)	16.1 (15.5–16.6)	<.001
Current feelings of anxiety	11.3 (10.9–11.8)	19.4 (16.4–22.7)	11.1 (10.6–11.5)	<.001
Ever diagnosed with depressive disorder	17.3 (16.8–17.9)	33.9 (30.4–37.6)	16.8 (16.3–17.4)	<.001
Current feelings of depression	4.5 (4.2–4.8)	11.1 (8.7–13.9)	4.3 (4.0–4.6)	<.001
Ever diagnosed with both anxiety and depression	11.0 (10.6–11.5)	20.3 (17.2–23.6)	10.7 (10.3–11.2)	<.001
Current feelings of both anxiety and depression	3.2 (2.9–3.4)	8.0 (6.0–10.4)	3.0 (2.8–3.3)	<.001
**Mental health care**				
Currently taking medication for anxiety	12.9 (12.4–13.4)	21.0 (18.1–24.2)	12.7 (12.2–13.2)	<.001
Currently taking medication for depression	10.6 (10.2–11.0)	20.3 (17.4–23.5)	10.3 (9.9–10.7)	<.001
Currently taking medication for both anxiety and depression	8.3 (7.9–8.6)	15.1 (12.5–18.1)	8.1 (7.7–8.4)	<.001
Received counseling in the past 12 months	11.1 (10.7–11.6)	13.7 (11.2–16.4)	11.1 (10.6–11.5)	.055
Currently receiving counseling	7.1 (6.7–7.5)	9.2 (7.1–11.7)	7.0 (6.7–7.4)	.06

a The National Health Interview Survey is conducted by the National Center for Health Statistics (15). All values are percentage (95% CI).

b *P* value from Wald *F* tests, testing for any differences in mental health characteristic between adults with kidney disease and without kidney disease.

In the multivariable model that adjusted only for sociodemographic characteristics, adults diagnosed with kidney disease remained almost 3 times more likely than adults not diagnosed with kidney disease to experience SPD and about 2 times more likely to have a diagnosis of anxiety disorder (APR = 1.74; 95% CI, 1.52–1.98), depressive disorder (APR = 1.97; 95% CI, 1.76–2.21), and both anxiety and depression (APR = 2.02; 95% CI, 1.72–2.39), and to have current symptoms of anxiety (APR = 1.94; 95% CI, 1.65–2.28) and depression (APR = 2.28; 95% CI, 1.78–2.91) and symptoms of both (APR = 2.26; 95% CI, 1.67–3.06) (Table 3). In the multivariable model for mental health care measures that adjusted only for sociodemographic characteristics, the PRs for taking medication for anxiety or depression and receiving counseling, both in the past 12 months or currently, remained significantly higher among adults diagnosed with kidney disease compared with those not diagnosed with kidney disease. After including health characteristics, the association between mental health status and care attenuated but remained significant for SPD, diagnosis of anxiety disorder, current feelings of anxiety, diagnosis of depressive disorder, diagnosis of both anxiety and depression, and receipt of counseling.

**Table 3:** Unadjusted and Adjusted Prevalence Ratio (PR) of the Association Between Diagnosed Kidney Disease and Selected Measures of Mental Health Status and Care, National Health Interview Survey, 2021^a^

Measure	Unadjusted PR (95% CI)	PR (95% CI) adjusted for sociodemographic characteristics^b^	PR (95% CI) adjusted for sociodemographic and health characteristics^c^
**Mental health status**			
Serious psychological distress	3.00 (2.33–3.86)	2.91 (2.23–3.81)	1.54 (1.16–2.06)
Ever diagnosed with anxiety disorder	1.63 (1.44–1.86)	1.74 (1.52–1.98)	1.18 (1.02–1.38)
Current feelings of anxiety	1.75 (1.49–2.06)	1.94 (1.65–2.28)	1.25 (1.03–1.52)
Ever diagnosed with depressive disorder	2.02 (1.81–2.24)	1.97 (1.76–2.21)	1.39 (1.21–1.61)
Current feelings of depression	2.61 (2.07–3.29)	2.28 (1.78–2.91)	1.29 (0.99–1.67)
Ever diagnosed with both anxiety and depression	1.89 (1.61–2.22)	2.02 (1.72–2.39)	1.29 (1.07–1.57)
Current feelings of both anxiety and depression	2.62 (1.98–3.47)	2.26 (1.67–3.06)	1.23 (0.90–1.68)
**Mental health care**			
Currently taking medication for anxiety	1.66 (1.43–1.92)	1.62 (1.38–1.89)	1.07 (0.90–1.28)
Currently taking medication for depression	1.97 (1.69–2.30)	1.82 (1.55–2.13)	1.19 (0.99–1.43)
Received counseling in the past 12 months	1.23 (1.02–1.50)	1.71 (1.42–2.05)	1.29 (1.04–1.59)
Currently receiving counseling	1.31 (1.03–1.67)	1.79 (1.4–2.28)	1.35 (1.02–1.77)

a The National Health Interview Survey is conducted by the National Center for Health Statistics (15).

b Model adjusted for sex, race and Hispanic origin, age, marital status, education, family income, and urbanization level.

c Model adjusted for sociodemographic characteristics listed in preceding footnote plus self-reported health status, and diagnosis of diabetes, coronary heart disease, hyperlipidemia, and hypertension.

## Discussion

This study examined mental health status and care among adults with and without diagnosed kidney disease by using nationally representative survey data for the civilian noninstitutionalized US adult population. This study found that the prevalence of experiencing symptoms of a serious mental illness in the past 30 days, as measured by the K6 scale for SPD, and currently experiencing feelings or symptoms of anxiety and depression that can affect functioning, based on questions from Washington Group on Disability Statistics, was higher among adults with diagnosed kidney disease than adults without diagnosed kidney disease. Adults diagnosed with kidney disease were more likely than adults not diagnosed with kidney disease to have ever been diagnosed with anxiety or depressive disorders. The percentage with coexisting symptomology of anxiety and depression and diagnosed comorbidity of these disorders was also higher among adults diagnosed with kidney disease than adults not diagnosed with kidney disease. This study did not investigate when adults were diagnosed with anxiety or depressive disorders. The finding that adults diagnosed with kidney disease were both more likely to have been diagnosed with anxiety or depressive disorders and currently experiencing symptoms of anxiety and depression that affect daily functioning suggests that, for some adults, a history of mental disorder diagnosis may have relevance to their present symptoms.

In multivariable analyses controlling for both sociodemographic and health characteristics, the association between diagnosed kidney disease and mental health status remained significant for SPD, current symptoms of anxiety, and diagnosed anxiety disorder, diagnosed depressive disorder, or both disorders. These findings support the comorbid nature of kidney disease and mental health conditions, namely anxiety and depression (6–11). The finding that current symptoms of depression and symptoms of both anxiety and depression were no longer significantly associated with diagnosed kidney disease when other health characteristics were included in the multivariable analyses supports that multiple comorbidities influence mental health symptoms. Among patients with predialysis kidney disease, illness perception (eg, “my kidney problem has major consequences on my life”, “my kidney problem will last for a long time”) has been associated with mental health distress (26). In cohort studies, depression has been associated with worsening kidney function (27,28).

This study also revealed a higher prevalence of use of mental health services among adults diagnosed with kidney disease compared with adults not diagnosed with kidney disease. The receipt of mental health counseling, currently and in the past 12 months, remained significantly associated with diagnosed kidney disease in multivariable analyses that controlled for both sociodemographic and health characteristics. This finding suggests that adults diagnosed with kidney disease are more likely than the general population to receive counseling for emotional support and well-being. A previous study found that among adults with kidney disease, those with more severe psychological distress were more likely than those with mild to moderate or no distress to have seen a mental health professional in the past year (29).

The prevalence of the use of prescription medication to treat anxiety and depression was higher among adults diagnosed with kidney disease compared with adults not diagnosed with kidney disease. However, these associations were no longer significant when accounting for differences in both sociodemographic characteristics and health status and other chronic conditions between adults with and without diagnosed kidney disease. Findings from a systematic review on the safety and efficacy of antidepressants in adults with renal impairment highlight the importance of adjusting doses, and customizing to the patient’s renal status and mental health symptoms, comorbidities, and potential drug interactions (30). Research in psychopharmacological interventions for adults with kidney disease is limited (30,31).

### Strengths and limitations

One strength of this study is that it presents nationally representative estimates of several self-reported mental health measures and key aspects of mental health care use, offering insights into the public health burden and management of mental health needs of US adults diagnosed with kidney disease. This analysis enhances our understanding of the intersection between diagnosed kidney disease and mental health conditions. The study also indicates that the increased prevalence of mental health symptoms such as SPD, anxiety symptoms, and diagnosis of depression and anxiety disorders were independent of additional high-mortality chronic comorbidities examined in the analyses.

This study has several limitations. This is a cross-sectional analysis, so the temporality of the associations between mental health status and care and kidney disease could not be ascertained. The NHIS asks a single interview question on whether the respondent was ever diagnosed with weak and failing kidneys, which limits the ability of this study to characterize the severity and duration of kidney failure and undergoing treatment status. This study does not offer data findings on adults who have decreased kidney function and are unaware of having this condition. While about 2.9% of adults were identified as having kidney disease in this study, it is estimated that about 14.0% of US adults have chronic kidney disease in stages 3a–5 when kidney function biomarkers are examined (32,33). Awareness of having kidney disease has consistently been found to be very low in the general population, and awareness increases with the number of manifestations of renal dysfunction (16,33,34). This differential misclassification of disease status may be confounded by interactions with the health care system and the opportunity, or lack of, to be diagnosed with a disease and receive further health care assessments, including mental health care. Furthermore, misclassification related to awareness of kidney disease status may result in either an underestimate or overestimate of the relationship between kidney disease and mental health symptoms, which segues into use of mental health care. For example, the observed relationships between diagnosed kidney disease and mental health symptoms and care could be underestimated when people unaware of having mild to moderate kidney disease are misclassified as not having kidney disease. Conversely, since awareness of kidney disease may be related to disease severity, and severity may be related to mental health symptoms in a bidirectional way, the observed association could be overestimated.

The pattern of diagnosed kidney disease by age and selected race and ethnicity in this study was consistent with estimates of kidney disease obtained from biomarker data (32,33). In the absence of additional disease information, biomarker data, and clinical data, the findings of this report are limited to noninstitutionalized adults who reported having been diagnosed with kidney disease and thus were aware of having this condition, and for whom disease severity, duration, and presence of kidney replacement therapy are unknown.

NHIS responses are self-reported by the sample adult respondent, unless unable to do so due to a physical or mental condition, in which case a proxy respondent responds for the selected adult. Information collected may be subject to recall bias, and responses on mental health symptoms may be considered sensitive to the respondent and subject to social desirability bias, and for the small percentage of proxy responses, the respondents may not be aware of or know the severity of mental health distress experienced by the sample adult. The inclusion of population-based validated diagnostic and severity measures of symptoms of depressive and anxiety disorders are included in the NHIS in years when the question on kidney disease is not asked, which limited the ability to assess these conditions. Unmeasured confounders such as substance use could not be accounted for in the analysis. NHIS collects information about alcohol use in years when the question on kidney disease is not asked, and it does not collect information on illicit drug use. Future analysis on this topic could examine tobacco use as a confounder. While this study examined the use of prescriptions for mental health and counseling, the NHIS does not include questions that assess whether the mental health care received is perceived to be sufficient or adequate to meet the sample adult’s needs.

### Conclusion

Analyzing mental health status and access to care among adults with known kidney disease across sociodemographic groups may provide valuable insights into potential differences. The NHIS data offer a unique opportunity to explore these patterns, which may guide future considerations in addressing the mental health needs of this population.

## Appendix

**Supplemental Table:** National Health Interview Survey Questions and Classification of Response Options

Measure	Question	Classification of response options
**Mental health status**		
Serious psychological distress based on the Kessler 6 scale (17,18)	*“*During the past 30 days, how often did you feel: 1) So sad that nothing could cheer you up; 2) Nervous; 3) Restless or fidgety; 4) Hopeless; 5) That everything was an effort; and 6) Worthless.”	The response option for each question is scored as follows: 0 for “none of the time,” 1 for “a little of the time,” 2 for “some of the time,” 3 for “most of the time,” and 4 for “all of the time.” Scores were summed to produce a total score from 0 to 24. A score of ≥13 was used to classify experiencing serious psychological distress.
Ever diagnosed with anxiety disorder	“Have you ever been told by a doctor or other health professional that you had a type of anxiety disorder?” Some common types of anxiety disorders include generalized anxiety disorder, social anxiety disorder, panic disorder, posttraumatic stress disorder, obsessive-compulsive disorder, and phobias.	Responses were yes and no.
Current feelings of anxiety disorder from Washington Group on Disability Statistics (19).	“How often do you feel worried, nervous or anxious? Would you say daily, weekly, monthly, a few times a year, or never?” “Thinking about the last time you felt worried, nervous or anxious, how would you describe the level of these feelings? Would you say a little, a lot, or somewhere in between?”	Adults who reported having the feelings daily and described the level of those feelings as “somewhere in between a little and a lot” or “a lot” or having the feelings weekly and described the level of those feelings as “a lot” were classified as having current feelings of anxiety.
Ever diagnosed with depressive disorder	“Have you ever been told by a doctor or other health professional that you had a type of depression?” Some common types of depression include major depression or major depressive disorder, bipolar depression, dysthymia, postpartum depression, and seasonal affective disorder.	Responses were yes and no.
Current feelings of depression from Washington Group on Disability Statistics (19)	“How often do you feel depressed? Would you say daily, weekly, monthly, a few times a year, or never?” “Thinking about the last time you felt depressed, how depressed did you feel? Would you say a little, a lot, or somewhere in between?”	Adults who reported having the depressed feelings daily and described the level of the feelings as “somewhere in between a little and a lot” or “a lot” or having the feelings weekly and described the level of the feelings as “a lot” were classified as having current feelings of depression.
Ever diagnosed with both anxiety and depression	See questions on ever been diagnosed with anxiety disorder and depressive disorder.	Combined responses to ever been diagnosed with anxiety disorder and with depressive disorder.
Current feelings of both anxiety and depression	See questions on current feelings of anxiety and depression.	Combined responses to current feelings of anxiety and depression.
**Mental health care**		
Currently taking medication for anxiety	“Do you take prescription medication for these feelings?” This question was asked of all adults after the question of how often they feel worried, nervous, or anxious.	Responses were yes and no.
Currently taking medication for depression	“Do you take prescription medication for depression?”	Responses were yes and no.
Currently taking medication for both anxiety and depression	See question on taking prescription medication for anxiety and depression.	Combined responses to taking prescription medication for anxiety and depression.
Received counseling in the past 12 months	“During the past 12 months, did you receive counseling or therapy from a mental health professional such as a psychiatrist, psychologist, psychiatric nurse, or clinical social worker?”	Responses were yes and no.
Currently receiving counseling	“Are you currently receiving counseling or therapy from a mental health professional?” Respondents who answered no to receiving counseling in the past 12 months are included in the denominator.	Responses were yes and no.
**Selected health characteristics**		
Kidney disease	“Have you ever been told by a doctor or other health professional that you had weak or failing kidneys?” Do not include kidney stones, bladder infections, or incontinence.	Responses were yes and no.
Diabetes	“Has a doctor or other health professional ever told you that you had diabetes?” Adult females who had previously reported gestational diabetes were asked not to include it when answering this question.	Responses were yes and no.
Coronary heart disease	“Have you ever been told by a doctor or other health professional that you had coronary heart disease?”	Responses were yes and no.
Hyperlipidemia	“Have you ever been told by a doctor or other health professional that you had high cholesterol?” “During the past 12 months, have you had high cholesterol?” and “Are you now taking any medication prescribed by a doctor to help lower your cholesterol?”	Responses were yes and no. Adults who responded yes to either of last 2 questions referencing the past 12 months were classified as having high cholesterol.
Hypertension	“Have you ever been told by a doctor or other health professional that you had hypertension, also called high blood pressure?” “During the past 12 months, have you had hypertension or high blood pressure?” and “Are you now taking any medication prescribed by a doctor for your high blood pressure?”	Responses were yes and no. Adults who responded yes to either of last 2 questions referencing the past 12 months were classified as having hypertension.
Self-reported health status	“Would you say your health in general is excellent, very good, good, fair, or poor?”	Excellent, very good and good versus fair and poor.
**Sociodemographic characteristics**		
Age	“What is your age?”	Age in years was categorized into 18-44, 45-64 and ≥65 years
Education	“What is the highest level of school you have completed or the highest degree you have received?”	Twelve response options were combined into 1) High school or less (never attended or kindergarten only, grade 1–11, 12th grade no diploma, GED or equivalent, high school graduate); 2) Associate's degree or some college (some college no degree, Associate degree: occupational, technical, or vocational program, Associate degree: academic program); and 3) Bachelor’s degree or higher (bachelor’s degree, master’s degree, professional school degree, or doctoral degree).
Family income	“What is your best estimate of [your total income/the total income of all family members] from all sources, before taxes, in [previous year]?” If two or more persons per family, the question was preceded with the statement “when answering this next question, please remember to include your income plus the income of all family members living in this household.” Respondents who did not provide a family income were asked a series of follow-up questions using a closed-ended income range and in relation to the federal poverty level. “Was your total family income from all sources less than [lower bound] or [upper bound] or more?” Family status and size of household are determined during household rostering. This report uses family income as a percentage of the federal poverty level (15). Family income was imputed when missing (21).	Categorized into less than 100% the federal poverty level, 100% to less than 200% federal poverty level, and 200% and greater the federal poverty level.
Marital status	Marital status is determined from a series of branching questions: “Are you now married, living with a partner together as an unmarried couple, or neither?” “Does your spouse live here?” “Which person is your spouse?” “Does your spouse not live here because you and your spouse are legally separated?” “Which person is your partner?” “Have you ever been married?” “What is your current legal marital status?” “Are you married, widowed, divorced, or separated?” and “Are you widowed, divorced, or separated?”	Adults were categorized into mutually exclusive categories: 1) married (and spouse present, not present or unknown in household); 2) widowed, divorced or separated; 3) never married; and 4) cohabiting or living with a partner.
Race and Hispanic origin	Combined responses to separate questions: “Do you consider yourself to be Hispanic or Latino?” and “What race or races do you consider yourself to be?”	Response to question of Hispanic origin were yes and no. Respondents had the option to select ≥1 racial group: Alaska Native, American Indian, Asian, Black or African American, Native Hawaiian, Pacific Islander, White, or some other race (with additional detail collected). Adults categorized as Hispanic could be of any race or combination of races, and adults categorized as non-Hispanic indicated one race only.
Sex	“Are you male or female?”	Male or female
Urbanization level	Derived from the location of the selected sample address and using the 2013 NCHS Urban–Rural Classification Scheme for Counties (22).	Large metropolitan area are counties (or county equivalents) in metropolitan statistical areas (MSA) of 1 million or more population; Medium or small metropolitan area are counties (or county equivalents) in MSAs of 250,000 to 999,999 population or in MSAs of less than 250,000 population; Nonmetropolitan area are counties (or county equivalents) in micropolitan statistical areas and non-core counties.
